# Alternative headphones for patient noise protection and communication in PET-MR studies of the brain

**DOI:** 10.1186/s13550-018-0457-6

**Published:** 2018-12-03

**Authors:** Lutz Tellmann, Hans Herzog, Frank Boers, Christoph Lerche, N. Jon Shah

**Affiliations:** Institute of Neuroscience and Medicine – 4, INM-4, Forschungszentrum Jülich, Leo-Brandt-Str., Jülich, 52425 Germany

**Keywords:** PET, MRI, PET-MR, Brain imaging, NeuroPET, Attenuation correction, MR headphones, Image artefacts

## Abstract

**Introduction:**

Due to the high noise emission generated by the gradients in magnetic resonance imaging (MRI), an efficient method of noise protection is mandatory. In addition to providing hearing protection, appropriate headphone systems also serve to facilitate communication between the operator and the patient. However, in combined PET-MR devices, use of common pneumatic headphones, as delivered by the manufacturer, is problematic due to the potential generation of attenuation artefacts in the PET measurement. Furthermore, modern multichannel head coils rarely provide space for conventional headphones. This work presents an alternative system, which aims to address these limitations while still being appropriate for both patient noise protection and communication in PET-MR.

**Material and methods:**

As an alternative to the standard headphones supplied with the PET-MR (3T MR-BrainPET, Siemens), the possibility of using earphones built out of commercially available earplugs has been investigated. The air channel (E-A-RLink) of the earplug is connected to the tubes of the original headphones. The attenuation characteristics of the conventional headphones and of the modified earphones were measured using a dedicated PET system with a ^68^Ge transmission source. For this purpose, the headphones, and then the earphones, were attached to a non-radioactive head phantom. To investigate the influence of the different phones on PET emission images, measurements of the head phantom, filled with ^18^F solution, were performed in the PET-MR. A measurement of the head phantom without headphones or earphones was used as a reference.

**Results:**

The linear attenuation coefficient of the headphones was 0.11 cm^-1^ and that of the head phantom 0.10 cm^-1^. The earphones were not identifiable in the transmission image. The emission image showed an activity underestimation of 10% near the headphones, compared to the reference image, whereas the earphones did not affect the image. Communication with the patient via the earphones was successful, and the noise protection—as confirmed by investigated subjects—was satisfying.

**Conclusion:**

The presented earphones, which can be connected to the existing patient communication system, are a preferable alternative to the conventional headphones, as, in contrast to the use of headphones, qualitative and quantitative errors in the PET images can be avoided. Patient acceptance of the earphones was high, despite the increase in preparation time before the PET-MR study.

## Introduction

Due to the high level of noise generated by the gradient coils in the strong magnetic field during a magnetic resonance imaging (MRI) examination, noise protection for the patient is mandatory. Furthermore, to allow the operator to remain in direct communication with the patient during the examination, it is necessary for the patient to be able to hear the operator. Noise protection and communication are typically realised with the use of non-magnetic headphones, which are driven by air instead of electric or electronic components to ensure MR compatibility. In addition to the constraint of MR compatibility, the decreasing space in newer multichannel head coils means that space-saving solutions for the headphones are required. The headphones must not be visible by the MR and must not influence the image quality of the MR measurement.

With the increasing use of combined PET-MR systems, without any facility for measuring the attenuation of radiation caused by all objects inside the field of view of the PET, MR-based attenuation correction has become an important issue. A number of methods have been suggested and are available for the attenuation correction of the head from patient to patient [1–4]. Other objects, such as RF coils, patient support, headphones, mirrors, spectacles, and EEC-electrodes, can cause supplemental attenuation, in addition to the attenuation by anatomy. While RF coils and patient support are stationary and can be considered by attenuation templates, the position of the other mentioned objects is variable, rendering corresponding templates useless. However, if the attenuation due to these objects cannot be neglected, the reconstructed PET images may deliver erroneous quantitative values in regions nearby the object or, in worse cases, corresponding artefacts that are visible in the images [5–8].

In this paper, we present customised earphones with very low gamma ray attenuation as an alternative solution to the manufacturer-delivered headphones.

## Material and methods

**Fig. 1:**
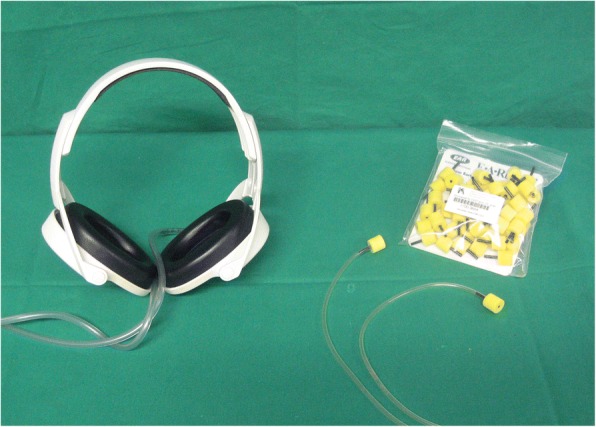
Left: standard pneumatic headphone (Siemens), right: earphones (3M) connected to the pneumatic audio system of the MR

The 3T MR-BrainPET prototype (BrainPET, Siemens Healthcare GmbH, Erlangen) [9, 10] used in this study was delivered with standard MR headphones made out of two plastic earcaps, connected to an air pressure-driven acoustic system to provide the sound to the patient’s ear. The acoustic signal is transmitted through a pneumatic device with plastic tubes to the earcaps (Fig. 1, left). There is no discrimination between the right and left ear, so the stereo presentation of the audio signal is not possible. The individual volume level can be adjusted subjectively from the console. As an alternative to the standard headphones, earphones built in-house from disposable/single-use foam earplugs (3M E-A-RLINK), connected to the pneumatic system via small standard PVC tubes, were applied (Fig. 1, right). According to the manufacturers’ specifications, the sound absorption in the standard headphone system is around 14 dB and for the earphone earplugs 30 dB.

**Fig. 2:**
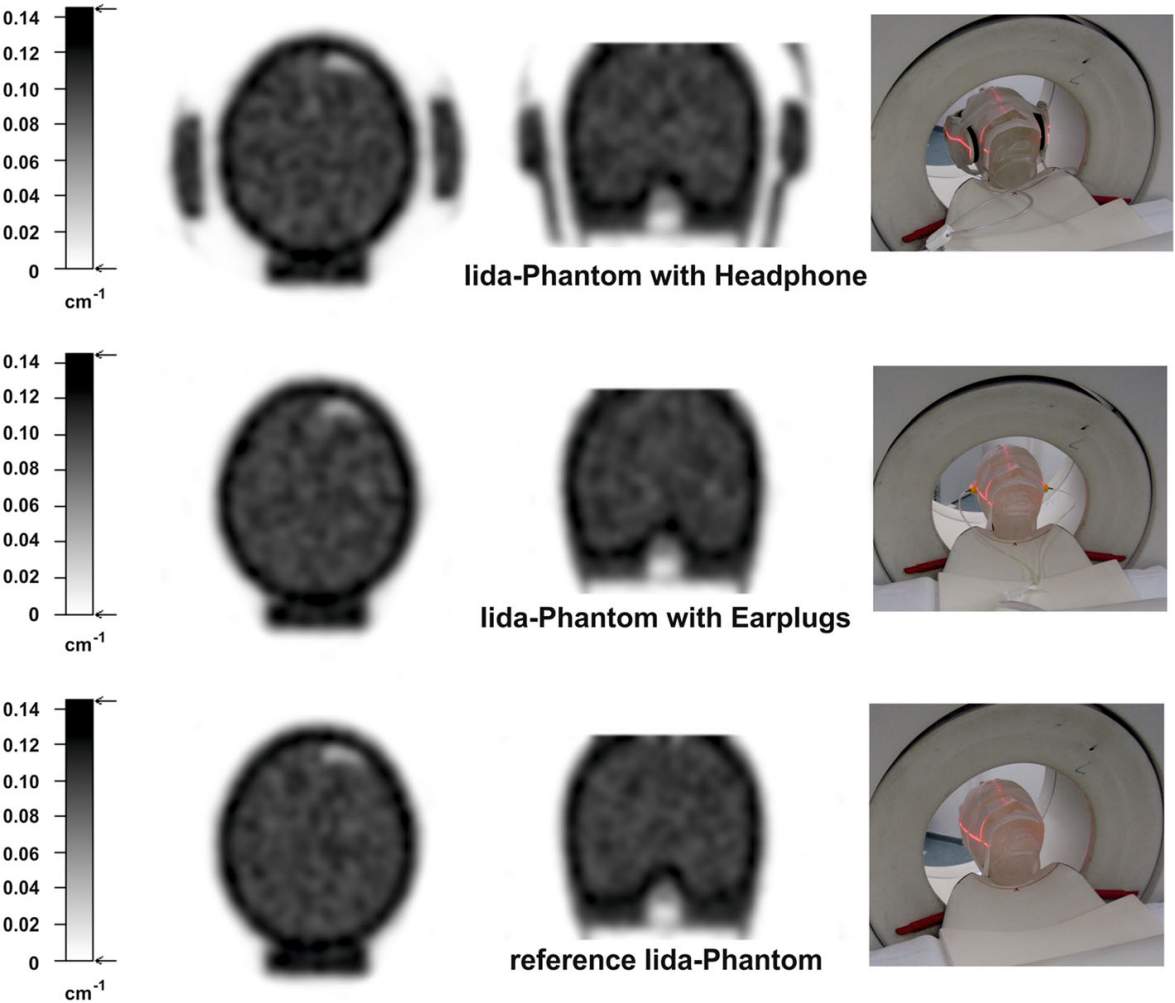
Transmission images of the Iida phantom with headphones, earphones and without any phones (reference)

Using a dedicated PET system ECAT Exact HR+ (Siemens/CPS), equipped with rotating ^68^Ge sources, the attenuation characteristics of the headphones, and then the earphones, were determined using 20 min transmission scans of the Iida brain phantom [11] without any radioactivity in any compartments. One scan was conducted with the mounted phones, where the earphones were taped onto the surface of the phantom using adhesive tape, and another scan without the mounted phones (Fig. 2). To avoid the need for an additional registration process in the analysis of the attenuation data, the phantom itself remained unmoved between the single measurements. All data sets were reconstructed with OSEM2D (6 iterations, 16 subsets) into transmission images, with a matrix of 256 × 256 and 63 slices, resulting in a pixel size of 2.0 × 2.0 mm^2^ and a slice thickness of 2.43 mm. The attenuation coefficients of headphones and the earphones were estimated by regional image analysis.

Emission measurements of the Iida brain phantom filled with ^18^F (~ 62.5 kBq/ml) in its grey matter compartment both with and without phones were performed in the BrainPET (Siemens) inside the MR (Siemens Tim Trio). During the PET measurement, different MR sequences were acquired. PET image reconstruction was obtained with OP-OSEM3D (32 iterations, 2 subsets). The resulting images consisted of 256 × 256 × 153 voxels, with a size of 1.25 mm in each direction. Attenuation correction of the emission data was done with both sets of the attenuation data acquired by the HR+ transmission measurement of the Iida phantom. The attenuation image was then spatially registered to the MR image and thereby to the emission data. The attenuation data of the Iida phantom were combined with the attenuation data of the coil [8]. Following a visual inspection of the emission images and difference images (headphones or earphones minus reference), the images were analysed with regions of interest (ROIs) located close to the phones inside the phantom, and the relative difference to the reference image without headphones or earphones was calculated.

In order to ascertain an impression of patient acceptance of the described earphones, fifty patients, mostly brain tumour patients with repeated measurements in different MR scanners, were asked to rate the comfort, loudness and total quality of communication with the earphones on a scale from − 2 to 2, where − 2 means very bad, 0 means neutral and + 2 excellent.

## Results

**Fig. 3:**
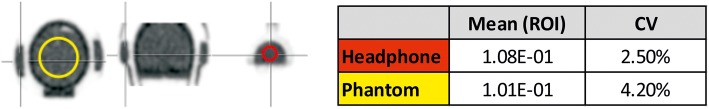
ROI analysis of the transmission images of the Iida phantom

The ROI analysis of the transmission images documents a mean attenuation coefficient of 0.101 cm^−1^ (coefficient of variance (CV) 4.2%) for the Iida phantom and 0.108 cm^−1^ (CV 2.5%) for the headphones. The earphones are not visible in the transmission image or in the difference image between the measurement with and without earphones (Fig. 3).

A visual inspection of the emission images shows no obvious artefacts in any of the images, also the acquired MR images showed no artefacts—either with or without phones. In the difference images between images with and without phones, an underestimation of up to 15% can be seen in the case of headphones, whereas no remarkable deviation can be found in the case of earphones.

**Fig. 4:**
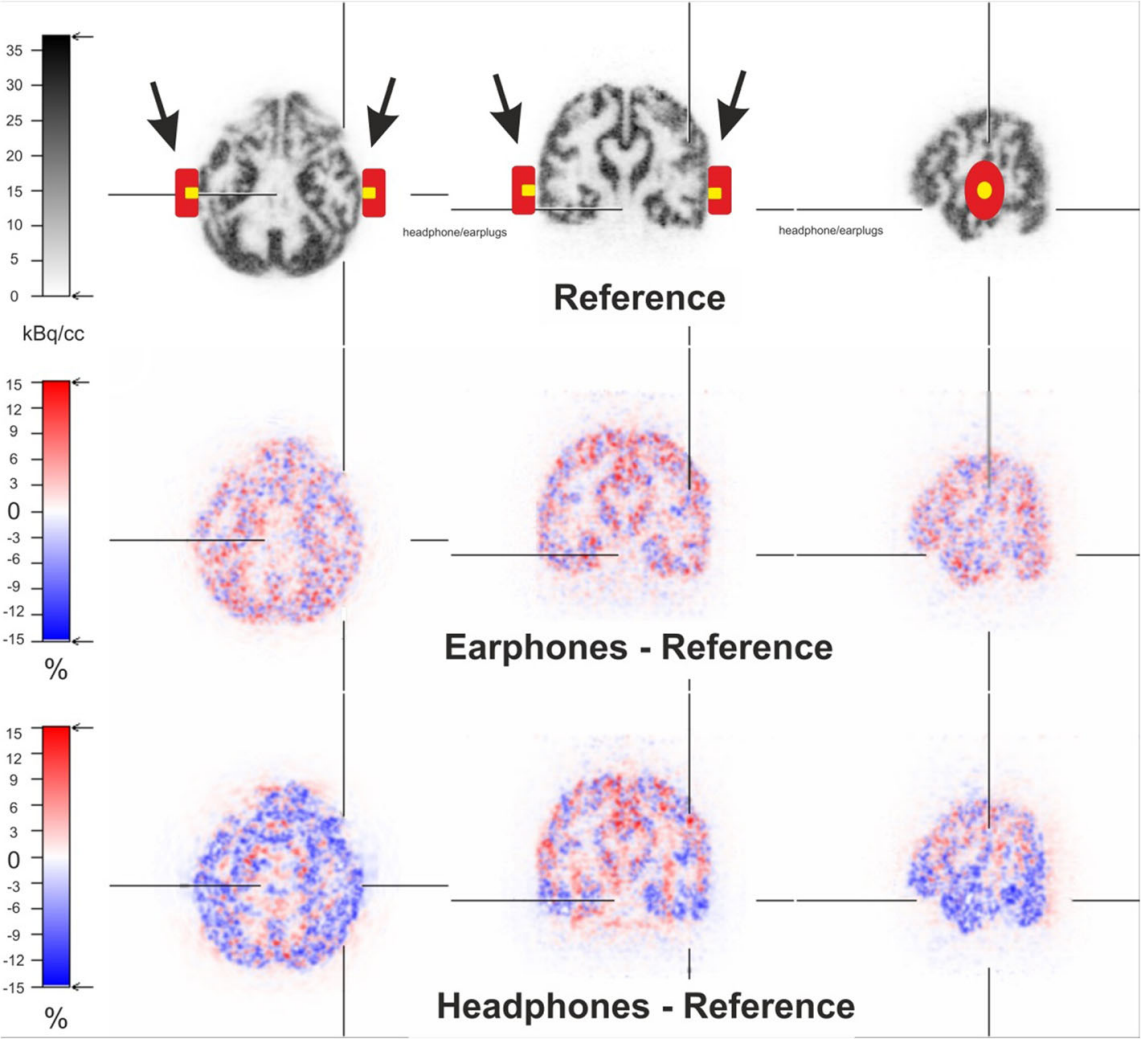
Emission image of the Iida phantom without headphones/earphones combined with the schematic drawing indicating the position of the phones if applied (above). Difference image between earphones and reference (middle) and between headphones and reference (below), respectively

The subsequent ROI analysis (Table 1) of the emission data with two elliptical ROIs placed inside the phantom nearby the headphone’s location results in an activity concentration of about 27.2 kBq/cc (CV 33%) on the right side and 23.2 kBq (CV 44%) of the head phantom without headphones (reference). The activity concentrations in the same ROIs on the emission images of the phantom scanned with headphones were lower by 10%, with values of 24.7 kBq/cc (CV 35%) on the right side and 21.0 kBq/cc (CV 45%) on the left side. In the case of the earphones, the ROI analysis shows no deviation (mean 0.8%) to the reference image, 27.0 kBq/cc (CV 33%) on the right side and 23.0 kBq/cc (CV 44%) on the left side. A third elliptical ROI in a central structure of the phantom shows nearly no deviation in either kind of device, compared to the reference (headphones 0.4%, earphones 1%). These results show that the headphones cause a local artificial reduction of the measured activity distribution nearby the headphones, while the earphones show no influence in the emission image (Fig. 4). Thus, we can document that PET images of the brain phantom study, and consequentially real brain measurements, are not influenced by the attenuation of the earphones.

**Table 1:** ROI analysis of the emission image. Drawn ROIs nearby the headphones location and results

	Phantom only (reference)		Phantom with headphones			Phantom with earphones		
	Mean (kBq/cc)	CV* (%)	Mean (kBq/cc)	CV* (%)	Deviation to reference	Mean (kBq/cc)	CV* (%)	Deviation to reference (%)
Ccentral ROI	24.6	37	24.5	37	0.4	24.2	36	1
Right ROI	27.2	33	24.7	35	10	27.0	33	0.7
Left ROI	23.2	44	21.0	45	10	23.0	44	0.9

*Coefficient of variation

**Fig. 5:**
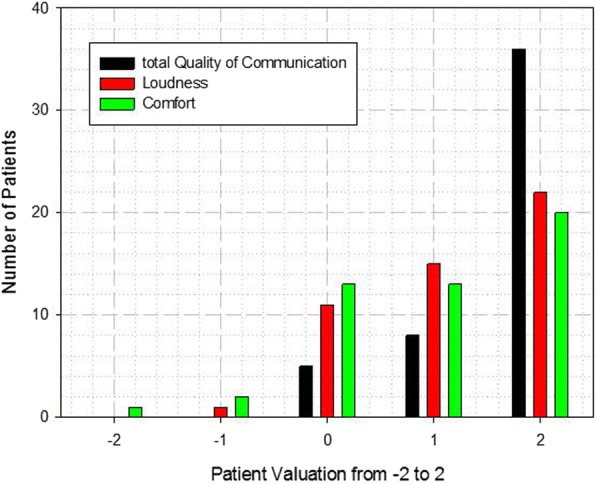
Result of the patient questionnaire (− 2, very bad; 0, neutral; + 2, excellent)

The questionnaire answered by the patients as an assessment for the earphones shows a high acceptance, despite the subjective higher effort required when inserting the earphones before the measurement. Most of the patients (89%) rated the total quality of the communication with the earphones with a positive grading (> 0 on a scale from − 2 to 2). Furthermore, the loudness, i.e. the reduction of the MRI noise, and the comfort were evaluated positively by most of the patients, with values of 76% and 68%, respectively (Fig. 5).

## Discussion

The earphones described and analysed in this report present a low-cost alternative solution to the typical manufacturer-provided headphones. They offer a favourable sound absorption and ensure communication with the subject. They show no local influence on reconstructed PET images in both qualitative and quantitative aspects and are not visible in the MR images. In contrast to the earphones, the standard headphones influence the quantification in the PET image in a range of about 10% caused by the wrong attenuation correction.

A template solution for the attenuation correction of the standard headphones is most difficult to realise because of the individual positioning of the different subjects and the complex determination of the position of the headphones. There have been a number of suggestions as to the best way to correct the attenuation caused by standard headphones. For example, Ferguson et al. [12] suggest a CT-derived attenuation map, and although the correction of the headphone attenuation was quite efficient, a residual attenuation effect of 1.9% remained. In contrast, Heußer et al. [13] based their attenuation correction of flexible hardware components, such as headphones, on a maximum likelihood reconstruction for attenuation and activity (MLAA). In phantom measurements, the authors found an underestimation of activity concentration of 13.4% without any correction and a maximum overestimation of 1.7% when applying the MLAA approach. For comparison, the use of the earphones presented here, which do not require any additional data processing, resulted in a quantitation error smaller than 1%.

For brain studies, and in particular quantitative and modelling studies, the earphones presented here should be the recommended system, as they combine favourable noise reduction of 30 dB and practical handling, despite the higher effort required for the patient to apply the earphones. An additional positive side effect is that these compact earphones offer an easier handling in the tight brain and head coils of MR systems. The earphones described here have been in use since 2009 when the MR-BrainPET system first came into use at the Forschungszentrum Jülich. Subsequently, an equivalent solution from Magnacoustic Inc., called MagnaCoils (http://www.magnacoustics.com/MagnaCoil.htm), has been introduced and is offered by allMRI GmbH and by Siemens. Since this system has a similar noise reduction as the in-house built earphones used by us and is considerably more expensive, we feel that there is no reason to replace our solution.

For audiometric studies, as performed in fMRI, the missing channel discrimination of the right and left ear and the inability to control the volume of the communication make the air-driven system unsuitable. For these applications, the air-driven system should be replaced by commercial, non-pneumatic earphone systems with a high-quality acoustic delivery and concurrent attenuation of the scanner noise (e.g. insert earphones S14 by Sensimetrics Corporation, Gloucester, MA, USA).

## Conclusion

The local biased image quantification induced by standard headphones in PET images, acquired during simultaneous PET-MR imaging, can be avoided by the use of low-cost disposable earphones. With the use of standard headphones, a visible influence on PET image quality and quantification is observed. In contrast, our study did not reveal measurable or visible influences in image quality or quantification when earphones made from disposable/single-use foam earplugs, connected to the pneumatic system via small standard PVC tubes, were used. Neither the headphones nor the earphones caused visible or measurable influence on MR image quality.

## Abbreviations

fMRI: Functional magnetic resonance imaging
MR: Magnetic resonance
MRI: Magnetic resonance imaging
PET: Positron emission tomography
